# Hidrocystome palpébral

**DOI:** 10.11604/pamj.2019.33.70.15601

**Published:** 2019-05-30

**Authors:** Fatima-Zahra Agharbi

**Affiliations:** 1 Hôpital Civil Tétouan Maroc, Centre Hospitalier Régional Tétouan, Maroc

**Keywords:** Paupières, hidrocystome, eccrine, appocrine, Eyelids, hidrocystoma, eccrine, appocrine

## Abstract

Palpebral hidrocystomas are benign tumors also known as cystic apocrine adenoma, cyst of sweat gland, apocrine retention cyst or cyst of Moll. They originate from eccrine or apocrine sweat glands and often occur on the face and the eyelids. Other atypical locations such as the chest, the shoulders and the foreskin have been reported. Hidrocystoma is a small translucent, shiny cyst. It appears as single or multiple cystic lesion. Their histogenesis is uncertain. Indeed, apocrine hidrocystoma could arise from the residuals of the primitive apocrine glands or of the gland of Moll. The eccrine hidrocystoma could arise from the eccrine glands or the excretory duct of the glands of Moll. Their clinical differentiation is little obvious and diagnosis is based on histological examination. They are characterized by two clinical presentations: isolated hidrocystomas and the associated types. Isolated hidrocystoma is the most common type: hidrocystoma appears as single or multiple cystic lesions and it is not associated with extraocular signs. Apocrine hidrocystomas are solitary in 93% of cases while eccrine hidrocystomas appear most often as multiple lesions. The associated types are more rare and have been only described for the multiple hidrocystomas. If in the majority of cases a treatment based on argon laser is sufficient, more voluminous hidrocystomas require surgical resection. We here report the case of a young patient presenting with translucent nodule in the external canthus of the left eye. Hidrocystoma, molluscum pendulum and syringoma were the suspected diagnoses. Surgical resection was performed as well as histologic examination which confirmed the diagnosis of hidrocystoma.

## Image en médecine

Les hidrocystomes palpébraux sont des tumeurs bénignes. Appelés également: adénome kystique apocrine, kyste sudoripare, kyste rétentionnel apocrine ou encore kyste de Moll, ils se forment aux dépens des glandes sudoripares eccrines ou apocrines. Souvent situés sur le visage et les paupières, d'autres localisations atypiques telles le thorax, les épaules et le prépuce ont été rapportées. L'hidrocystome réalise un petit kyste translucide, brillant, unique ou multiple. Leur histogenèse demeure discutée. En effet, l'hidrocystome apocrine pourrait dériver des résidus des glandes apocrines primitives ou de la glande de Moll et l'hidrocystome eccrine des glandes eccrines ou du segment excréteur des glandes de Moll. Leur différenciation clinique étant peu évidente, le diagnostic est histologique. Deux formes cliniques peuvent être caractérisées, les formes isolées et les formes associées. La forme isolée est la plus fréquente: l'hidrocystome est unique ou multiple et non associé à des signes extra-oculaires. Les hidrocystomes apocrines sont uniques dans 93% des cas alors que les hidrocystomes eccrines apparaissent le plus souvent multiples. Les formes associées sont plus rares et décrites uniquement pour les hidrocystomes multiples. Si dans la majorité des cas un traitement par laser Argon est suffisant, les hidrocystomes plus volumineux nécessitent une exérèse chirurgicale. Nous rapportons l'observation d'une jeune patiente qui consultait pour un nodule translucide du canthus externe de l'œil gauche. Les diagnostics évoqués étaient: un hidrocystome, un mollusum pendullum et un syringome. Une exérèse chirurgicale a été réalisée avec étude hiostologique confirmant le diagnostic d'hidrocystome.

**Figure 1:**
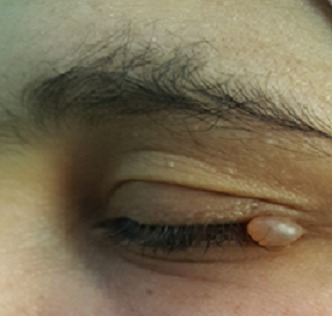
Nodule translucide du canthus externe de l’œil droit

